# Combined In Situ Hybridization and Immunohistochemistry on Archival Tissues Reveals Stromal microRNA-204 as Prognostic Biomarker for Oral Squamous Cell Carcinoma

**DOI:** 10.3390/cancers13061307

**Published:** 2021-03-15

**Authors:** Saroj Rajthala, Harsh Dongre, Himalaya Parajuli, Anjie Min, Elisabeth Sivy Nginamau, Arild Kvalheim, Stein Lybak, Dipak Sapkota, Anne Christine Johannessen, Daniela Elena Costea

**Affiliations:** 1 Gade Laboratory for Pathology, Department of Clinical Medicine, Faculty of Medicine, University of Bergen, N-5020 Bergen, Norway; saroj.rajthala@uib.no (S.R.); harsh.dongre@uib.no (H.D.); himalaya.parajuli@uib.no (H.P.); elisabeth.nginamau@uib.no (E.S.N.); anne.johannessen@uib.no (A.C.J.); 2 Centre for Cancer Biomarkers (CCBIO), Faculty of Medicine, University of Bergen, N-5020 Bergen, Norway; 3 Department of Oral Maxillofacial Surgery, Xiangya Hospital, Central South University, Changsha 410083, China; william0732@csu.edu.cn; 4 Department of Pathology, Haukeland University Hospital, N-5021 Bergen, Norway; 5 Oral Surgery Private Referral Practice “Tannteam”, N-5221 Nesttun, Norway; post@tannteam.no; 6 Head and Neck Clinic, Haukeland University Hospital, N-5021 Bergen, Norway; stein.lybak@helse-bergen.no; 7 Institute of Oral Biology, Faculty of Dentistry, University of Oslo, N-0316 Oslo, Norway; dipak.sapkota@odont.uio.no

**Keywords:** mir204, oral cancer, stroma, biomarker, chromogen, in situ hybridization, immunohistochemistry

## Abstract

**Simple Summary:**

In addition to the transformation of epithelial cells, dysfunction of stroma is crucial in carcinogenesis; cancer-associated stroma can regulate the phenotype of cancer cells and thereby influence the clinical outcome. Our study aimed to investigate the correlation of stromal miR-204 with progression of oral squamous cell carcinoma (OSCC) and assert its clinical utility. We first established a chromogen-based method that combined immunohistochemistry and in situ hybridization for exact delimitation of stroma from the tumor islands and concomitant visualization of miRs, and have developed a guide to digital miR quantification using the publicly available tool ImageJ and the licensed software Aperio ImageScope. We have then applied the method for investigating stromal miR-204 as a putative prognostic biomarker on an OSCC cohort and identified expression of miR204 in the stroma at tumor front as an independent prognostic biomarker for this disease.

**Abstract:**

Micro-RNAs (miRs) are emerging as important players in carcinogenesis. Their stromal expression has been less investigated in part due to lack of methods to accurately differentiate between tumor compartments. This study aimed to establish a robust method for dual visualization of miR and protein (pan-cytokeratin) by combining chromogen-based in situ hybridization (ISH) and immunohistochemistry (IHC), and to apply it to investigate stromal expression of miR204 as a putative prognostic biomarker in oral squamous cell carcinoma (OSCC). Four different combinations of methods were tested and ImageJ and Aperio ImageScope were used to quantify miR expression. All four dual ISH-IHC methods tested were comparable to single ISH in terms of positive pixel area percentage or integrated optical density of miRs staining. Based on technical simplicity, one of the methods was chosen for further investigation of miR204 on a cohort of human papilloma virus (HPV)-negative primary OSCC (*n* = 169). MiR204 stromal expression at tumor front predicted recurrence-free survival (*p* = 0.032) and overall survival (*p* = 0.036). Multivariate Cox regression further confirmed it as an independent prognostic biomarker in OSCC. This study provides a methodological platform for integrative biomarker studies based on simultaneous detection and quantification of miRs and/or protein and reveals stromal miR204 as a prognostic biomarker in OSCC.

## 1. Introduction

In addition to the transformation of epithelial cells, dysfunction of stroma is crucial in carcinogenesis [1,2]. The abnormal stroma surrounding carcinoma cells is referred to as reactive tumor stroma or cancer-associated stroma. Cancer-associated stroma comprises non-cancer cell constituents including cancer-associated fibroblasts, immune cells, adipocytes, endothelial cells, pericytes, nerves, extracellular matrix and secretomes synthesized by cells [1,3]. Cancer-associated stroma can regulate the phenotype of cancer cells and thereby influence the clinical outcome [1].

Better understanding of the role of cancer-associated stroma in carcinoma progression and therapeutic outcome has led to an increasing interest for stromal biomarkers in recent years. With the emergence of micro-RNAs (miRs) as important players in carcinogenesis [4,5,6,7], the hunt for new biomarkers has expanded to cancer-specific miRs in several biological specimens including blood [8,9], saliva [10], urine [11], stool [12], and tumors [13]. miRs control gene expression by targeting mRNA for cleavage or translational repression using a protein complex known as RNA-inducing silencing complex [7,14]. Several miRs have been found to promote or suppress cancer progression, and thereby are called oncogenic or tumor suppressor miRs [4,5,6,7]. 

As an example of putative tumor suppressor miR, miR-204 expression has been shown to decrease in several cancers [15,16,17,18,19]. In favor of its role as a tumor suppressor there are several studies showing anti-tumorigenic effects of miR-204 in both in vitro and in vivo animal studies [15,16,17,18,19,20,21]. Decreased expression of miR-204 has been associated with poor survival in breast cancer [17], gastric cancer [16], endometrial cancer [19], acute myeloid leukemia [22], medulloblastoma [20], and neuroblastoma [23]. Expression of miR-204 in neuroblastoma cells and gastric cancer cell lines increased their sensitivity to cisplatin [23], 5-fluorouracil, and oxaloplatin, respectively [16]. These studies support the notion that expression of miR-204 in cancer cells could be used both as prognostic marker and for targeted treatment. The same trend of decreased miR-204 expression in cancer cells with disease progression has been also described for oral squamous cell carcinoma (OSCC) [24,25,26]. Decreased expression of miR-204 in tumor cells was associated with increased lymph node incidence [27] and increased distant metastasis [26] in animal models of OSCC. Similarly, lowered miR-204 expression was shown to predict poor survival in OSCC [27]. 

Nevertheless, despite these studies on the expression and role of miR-204 in several cancers, including OSCC, there is a complete gap of knowledge in miR-204 function in the tumor stroma of carcinomas, including OSCC. All the above-mentioned studies were based on qRT-PCR, microarray, and sequencing techniques and were thus conducted selectively on cancer cells or on whole tumor tissues, which eludes the spatial distribution and regulation of miR-204 in different tumor compartments. Therefore, our study aimed to investigate the correlation of stromal miR-204 with OSCC progression and assert its clinical utility. 

Since single and poorly differentiated invading carcinoma cells are difficult to recognize without specific markers, thus leading to false positive results by erroneously being included in the quantification of the stroma, we aimed firstly to establish robust methods of combining miR in situ hybridization (ISH) with immunohistochemistry (IHC) for epithelial markers, e.g., pan-cytokeratin (pan-CK), for a more precise quantification of miRs in specific tumor compartments. We chose two oncogenic miRs, miR-21 and miR-155, for establishing the method since they were previously reported in the literature to have a biological relevance for OSCC and had different expression patterns which we thought would help in evaluating the double staining method we wanted to establish. Both miR-155 [28,29] and miR-21 [13] were shown to be overexpressed in OSCC tissues and were previously found to predict poor prognosis. Previous studies by single ISH found that miR-21 was primarily expressed in the tumor stroma and in some tumor-associated blood vessels with no expression in the adjacent normal epithelia or stroma [13], while miR155 was reported to be located in the cancer nests, inflammatory area, and vascular endothelium [28]. Here, we present robust ISH-IHC combination methods and a guide to digital miR quantification using the publicly available tool ImageJ and the licensed software Aperio ImageScope. By applying them to a well-annotated OSCC cohort of patients we show that stromal expression of miR-204 at the tumor front is an independent prognostic biomarker in OSCC. 

## 2. Materials and Methods

### 2.1. In Situ Hybridization (ISH)

ISH was performed following a modified protocol, while adhering to the staining principles in the Instruction Manual v3.0 (Exiqon A/S Vedbæk, Denmark). In brief, 3 µm formalin fixed paraffin embedded (FFPE) tissue sections were deparaffinized in xylene and rehydrated in a series of decreasing alcohol concentrations. To expose miR probes, we incubated tissue sections with 15 μg/mL Proteinase K solution (90,000; Exiqon) at 37 °C for 10 min, after a titration experiment that established the optimal unmasking treatment while maintaining tissue morphology. Tissues were then pre-hybridized with ISH buffer (90,000;) for 30 min, and then hybridized with locked nucleic acid-based digoxigenin (DIG)-labeled miR binding oligonucleotides at corresponding hybridization temperatures. miR-155-5p (619862-360; Exiqon) was hybridized at 48 °C; miR-21-5p (619870-360; Exiqon) and miR-204-5p (619857-360; Exiqon) were hybridized at 53 °C for 2 h. Optimal hybridization temperature for the individual miR target probes was determined using melting temperature-based temperature series test, and optimal concentration was determined using series of miR target probe concentration. Tissues were then washed stringently in decreasing concentrations of saline-sodium citrate (SSC) buffer (S66391L; MilliporeSigma, Munich, Germany) at corresponding hybridization temperatures. Following stringent wash, tissues were blocked in 2% sheep serum (013-000-121; Jackson ImmunoResearch, West Grove, PA, USA) and 1% bovine serum albumin (BSA). Tissues were then incubated with alkaline phosphatase (ALP)-linked anti-DIG Fc fragments (11093274910; Roche, Basel, Switzerland) at 1:400 concentration overnight at room temperature (RT). The following day, tissues were incubated with ALP substrate-Nitro blue tetrazolium chloride/5-Bromo-4-chloro-3-indolyl phosphate (NBT-BCIP) (11681451001; Roche) at 30 °C for 2 h or at RT overnight. The reaction was stopped using KTBT buffer, counterstained with nuclear fast red and mounted in xylene-based medium Pertex (00871.1000-EX; Histolab Products AB, Västra Frölunda, Sweden). Levamisole (X3021; Agilent Dako, Santa Clara, CA, USA) was used to block endogenous ALP activity. No probe and scramble oligonucletotide were used as negative controls; small nuclear RNA-U6 was used as positive control. 

### 2.2. Immunohistochemistry (IHC)

FFPE tissue sections were deparaffinized, rehydrated, antigen retrieved using Proteinase K, and blocked in sheep serum and BSA solution as described above in ISH section. p16INK^4a^ antigen was retrieved by boiling tissue sections in Tris-EDTA (pH 9) in a microwave oven for 15 minutes. Thereafter, tissue sections were incubated with monoclonal mouse anti-human primary antibody (pan-CK 1:800, Clone MNF116, Agilent Dako; p16INK4a 1:1000, G175-405, BD Pharmingen, NewYork, NJ, USA) at RT for 1 h. A Dako Envision+ System-HRP (DAB) kit (K4007; Agilent Dako) was used for the subsequent steps. Tissue endogenous peroxidase activity was blocked with peroxidase block for 5 min. Thereafter, sections were incubated with horseradish peroxidase (HRP)-conjugated secondary antibody for 30 min and visualized with diaminobenzidine (DAB) substrate at RT. Tissues were counterstained with fast red and mounted with Pertex. 

### 2.3. Combined miR ISH and IHC staining 

**Figure 1:**
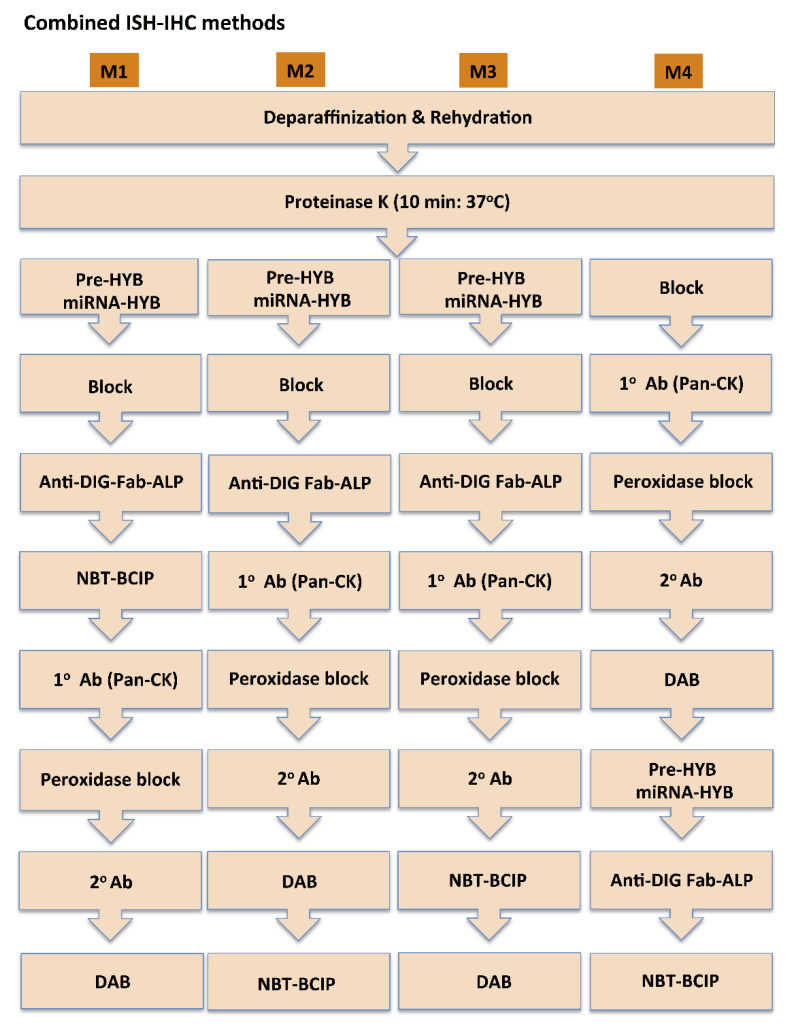
Flowchart depicting the ISH-IHC (in situ hybridization with immunohistochemistry) combinations employed in the double staining method.

ISH of miR and IHC of pan-CK were performed on the same tissue section following the methods described above, and in different order sequences as illustrated in the flowchart (Figure 1). 

### 2.4. Image Acquisition and Quantification

Images for the stained tissues were acquired at 40× objective using a whole slide scanner (Hamamatsu NanoZoomer-XR, Shizuoka, Japan). RGB vectors for NBT-BCIP, DAB and fast red were acquired using images from tissues stained with individual dye using “From ROI” interactive option in the Color Deconvolution plugin for ImageJ. Acquired RGB vectors for the stains were then integrated into Java for Color Deconvolution. Images were then color deconvoluted to resolve miR stain (NBT-BCIP) from fast red and DAB (Figure 1). Color threshold for NBT-BCIP was set to 195 to exclude background, and thereafter, positive pixel area percentage (PPAP) and integrated optical density (IOD; normalized to analysed area) of miR staining were measured. Mean pixel intensity was converted to OD using the function OD = log10 (255/mean pixel intensity); IOD was obtained as product of OD and positive pixel area stained and normalized to region of interest. The same measurements for the miR staining were made using other software, AperioI (Leica Biosystems, Wetzlar, Germany), using the same principles and criteria used in ImageJ. PPAP and IOD were quantified in the stroma regions of tumor center and tumor front of approximately 0.4–0.8 mm^2^. Five to seven hot spots, i.e., areas with highest staining intensity, were chosen for the quantification. Blood vessels, glands, muscles, and nerves were excluded from the study. For the methodological comparisons, similar regions in the tissue sections stained by different combination methods were chosen. MiR-21 and miR-204 were quantified both at tumor center and invasive tumor front. The invasive tumor front was defined as a 100 µm broad tissue area around the outermost invasive tumor islands.

### 2.5. Study Cohort 

The study cohort consisted of patients older than 18 years with primary diagnosis of OSCC between 1998 and 2012 and surgically, radio-, or combinatory treated at Haukeland University Hospital, Bergen, Norway (*n* = 169). Patients with neoadjuvant treatment, missing tissue blocks, and missing clinical information were excluded from the study. The clinical information (age, gender, smoking and alcohol use, localization, TNM stage, co-morbidities, recurrency, last date of follow-up, survival) was obtained from patients’ medical electronic journals and is presented in Table S1. FFPE tissue blocks containing the tumor front with surrounding stroma and adjacent normal human oral mucosa (NHOM) were selected for the study. Serial sections of 3–4 µm thickness were cut using a microtome (HistoCore Biocut, Leica Biosystems), mounted on glass slides (Superforst Plus from Thermo Fisher Scientific, Waltham, MA, USA), fixed on slides by incubation at 58 °C for 2 hours and stored at 4 °C until use. RNA contamination was avoided during cutting and handling using gloves and RNase decontaminating solution (RNase Zap, Thermo Fisher, Scientific). Tumor specimens were screened for human papillomavirus (HPV) infection with the surrogate marker p16INK4a by IHC. Nine cases (5.53%) displaying strong nuclear and cytoplasmic staining in more than 70–80% of the tumor cells and were excluded from the study. Finally, a total of 160 HPV-negative OSCC cases (age range: 27–93; mean = 65.25; median = 65) were included in the study. The mean follow-up time was 8.6 years, and the 5-year survival rate was 40%. Adequacy in sample size for the Cox regression was met as suggested by Peduzzi et al. [30]. NHOM from clinically healthy donors was also collected during wisdom tooth extraction (*n* = 14). Informed consents were obtained for the research use of tissues and clinical data. This study was approved by the regional ethical committee in Norway (REKVest 3.2006.2620 REKVest 3.2006.1341) and followed REMARK criteria [31]. 

### 2.6. Evaluation of Clinical and Pathological Parameters

Staging of OSCC (TNM) was done at the point of diagnosis according to the American Joint Committee on Cancer manual 6th edition. Tumor depth of invasion (DI), which is the distance from a theoretically reconstructed normal mucosal line to the deepest invasion point [32], and tumor budding (TB), which is defined as a single cell or a cluster of less than five cancer cells, were evaluated as described earlier [33]. Worst pattern of invasion (WPI) was scored as described by Brandwein-Gensler et al. [34]. Histological scoring was performed on scanned pan-cytokeratin stained images by an experienced pathologist (E.S.N). 

### 2.7. RNA Extraction and Quantitative Reverse-Transcriptase Polymerase Chain Reaction (qRT-PCR)

Approximately 10% of cohort samples (16 samples) were randomly selected and used for validation of miR-204 expression by using qRT-PCR. Three to four 10 μm freshly cut sections from FFPE samples were collected in RNase-free microtubes and RNA was isolated using miRNeasy FFPEkit (Qiagen, Oslo, Noway) according to the manufacturer’s guidelines. The paraffin around tissues was trimmed prior to sectioning, and the sections were macrodissected in order to select mainly the tumor front, removing the bulk of the tumor center or the normal surrounding tissue. Briefly, each sample was treated with deparaffinization solution, to remove excess paraffin. This was followed by mixing with buffer PKD and Proteinase K digestion with heat treatment (at 56  °C for 15 min and at 80 °C for 15 min). The DNA/RNA phases were separated by centrifugation at 20,000 *g* for 15 min. To further purify RNA, DNase treatment was applied to remove genomic DNA contamination. Each sample was then mixed with ethanol and transferred onto RNeasy MinElute spin columns. Following the manufacturer’s protocol of washing with RPE buffer, the RNA was subsequently eluted in 15 µL RNase-free water. RNA concentrations were measured using NanoDrop 1000 (Thermo Fisher Scientific). The RNA yield varied from 50 to 1800 ng/µL and the purity ranged from 1.7 to 1.99 (A260/A280 ratio).

Three RT reactions were performed for each sample (with 200 ng of input RNA for each reaction) in a 15 µL reaction using the TaqMan microRNA reverse transcription kit (Applied Biosystems, Foster City, CA, USA). RT primers were specific for each of the following miR: RNU6B (assay ID: 001093), RNU48 (assay ID: 001006), and hsa-miR-204 (assay ID: 000508). qRT-PCR reactions were performed on cDNA products and thus obtained with TaqMan Fast Advanced master mix II (Applied biosystems) according to the manufacturer’s protocol. PCR reactions were performed on a 7500 Fast Real-time PCR system (Applied Biosystems), with each reaction run in duplicates.

For analysis, the expression level of miR-204 was normalized to the mean of internal controls (RNU6B and RNU48) and the relative difference (ΔCt) was correlated with quantified ISH values (PPAP). Pearson correlation plot was used to establish significance and the data are represented as QQ plots (GraphPad Prism, v9, GraphPad Software, San Diego, CA, USA). 

### 2.8. Statistical Analysis 

Survival functions (overall survival (OS) and recurrence-free survival (RFS)) of clinicopathological parameters, including miR-204 expression in the tumor stroma, were plotted using the Kaplan–Meir (KM) method. Test of equality for survival distribution of the variables within the parameters was carried using the log-rank test (Mantel–Cox). Risks of clinicopathological parameters in the OS and RFS of the OSCC patients were further examined by univariate survival analysis using Cox’s proportional regression. Parameters with variables that exhibited significant risk difference in the OS and RFS in the univariate analysis were entered into the multivariate model to examine the risk adjusted to confounding variables. The proportional hazard assumptions, i.e., if the baseline hazard function was proportional or not, was checked graphically for all parameters with Log minus log function plot before regression analysis. Additionally, time-dependent covariates were modeled to check the proportionality of hazards over time (*p* < 0.05 indicates change of predictor over time). Tests of independence of clinicopathological parameters with miR-204 expression were assessed using Pearson’s chi-squared test. Kolmogorov–Smirnov test, Shapiro–Wilk test, histogram, and Q–Q plots were used to test the normality for the miR expressions. Tumor stroma expressions of miR-204 in the tumor front and tumor center were categorized into higher and lower expression group by the median expression value. Mann–Whitney U or Wilcoxon match-paired signed rank test was performed to find significant differences in miR in between high and low expression groups. Paired Student’s t-test was conducted to detect significant differences in mean of the PPAP and IOD for the miR expression in between control (single miR ISH) and double staining methods. The statistical analysis was performed using GraphPad Prism Version 7.0 (GraphPad Software) or IBM SPSS Statistics Version 25.0 (IBM Corp, Armonk, NY, USA). 

## 3. Results

### 3.1. miRs Expression and Their Co-Localization with pan-CK

**Figure 2:**
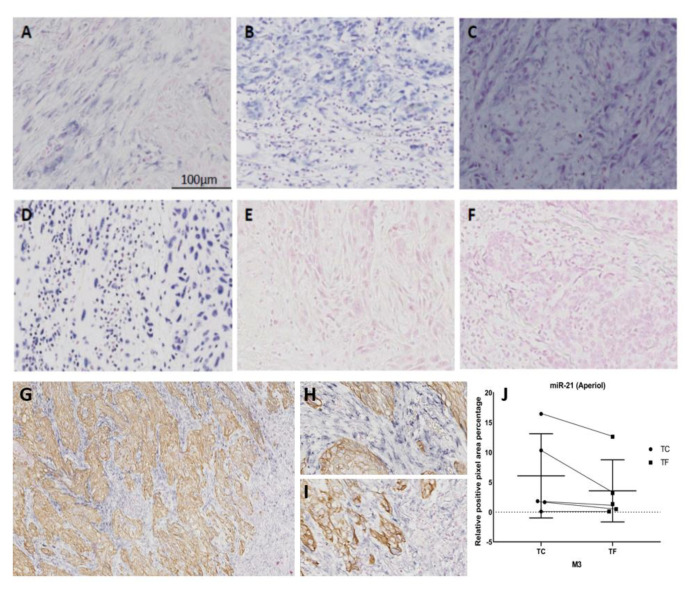
Representative images of ISH for various miRs: single ISH for miR-21 (**A**), miR-155 (**B**), and miR-204 (**C**); U6—positive control (**D**), scramble oligonucleotide with no target site—negative control (**E**), negative control without miR-binding probe (**F**), double ISH-IHC for miR-21 and pan-CK using method 3 (**G**) showing intense staining at tumor center (**H**) and weaker staining at tumor front (**I**) as quantified using AperioI in (**J**). Original magnification: 10×; scale bar: 100 µm.

Diverse spatial distribution of the expression of the investigated miRs was observed in OSCC tissues. miR-21 staining was confined to reactive tumor stroma (Figure 2), with higher staining intensity at tumor center compared to tumor front (Figure 2). The staining was observed almost exclusively in the cytoplasm of cells with a fibroblast-like appearance. Expression of both miR-155 (Figure 2) and miR-204 (Figure 2) was both epithelial and stromal; in the stroma it was localized both in fibroblast-like cells and lymphocytes. The abundance of the lymphocytic infiltrate varied from case to case, as shown in Figure 2 which depicts cases with poor (A,E), intermediate (C,F), and intense (B,D) lymphocytic infiltrate. Negative controls did not give any color signal (Figure 2). At 48 °C, scramble oligonucleotides nonspecifically bound to the tissue, and hence could not be used as negative control for miR-155 that hybridizes at 48 °C. 

**Figure 3:**
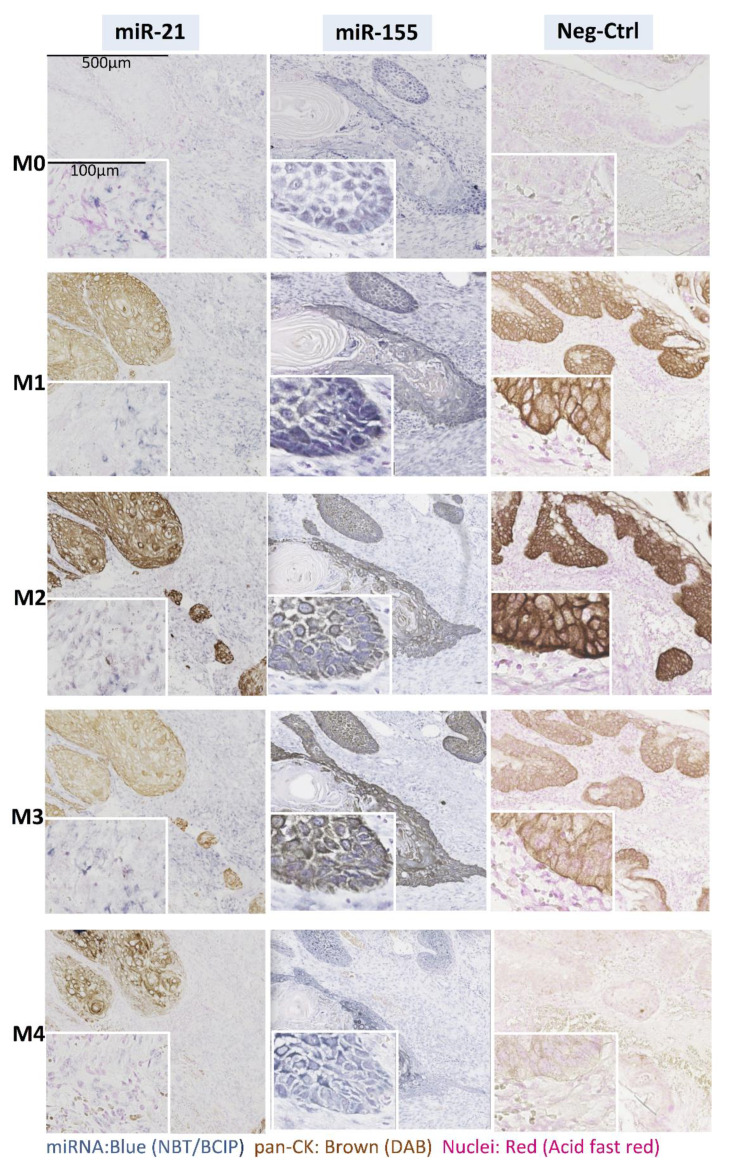
Representative images for combined miR ISH (miR-21 and miR-155) and IHC (pan-CK). Serial sections of the tissue were stained for miR alone (M0.ISH) or miRs ISH was combined with IHC of pan-CK (M1.ISH+IHC; M2.ISH-Fab+IHC+NBT-BCIP; M3.ISH-Fab+1oAb-2oAb+NBT-BCIP+DAB. M4. IHC+ISH). Scramble negative control or no probe control (Neg-Ctrl) was run for miR-21 and miR-155, respectively. Since no differences were observed, only scramble negative controls are presented. Original magnification: 5×; scale bar: 500 µm. Inset: original magnification: 20×; scale bar: 100 µm.

**Figure 4:**
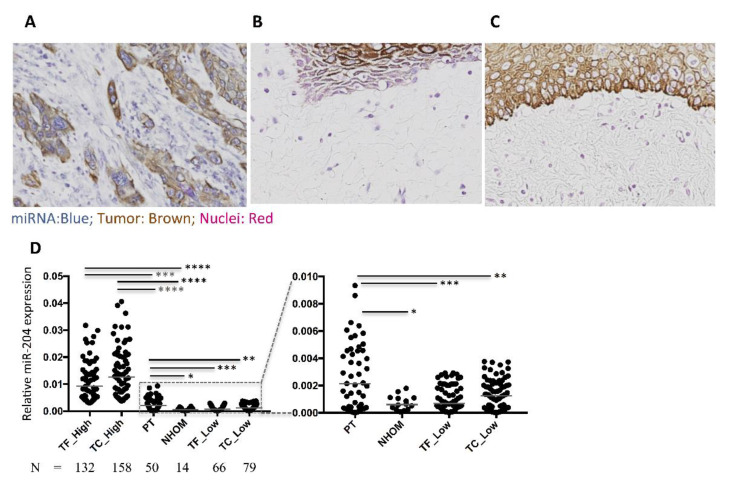
Representative images from a double stained section for miR-204 and pan-CK in tumor (**A**), peritumor (**B**), and normal mucosa (**C**) in OSCC. Original magnification: 20×; scale bar: 100 µm. (**D**) Wilcoxon test for the paired variables (tumor front (TF), tumor centre (TC), and peritumor (PT)). Mann–Whitney U test for all other comparisons (independent). **** *p* < 0.0001; *** *p* < 0.0005; ** *p* < 0.001; * = *p* < 0.05.

The epithelial marker pan-CK was specific to tumor cells in all double-stained tissue sections, as expected. No co-localization between pan-CK and miR-21 was observed, in line with previous studies that reported expression of miR21 exclusively in the tumor stroma of carcinomas, including OSCC [13]; however, both miR-155 (Figure 3) and miR-204 (Figure 4) colocalized with pan-CK in the epithelial islands. No nonspecific anti-DIG Fab binding or non-specific NBT-BCIP reaction to alkaline phosphatase was detected in negative controls run with scramble negative controls or without oligonucleotide probes.

### 3.2. Effects of IHC on ISH and Vice Versa

**Figure 5:**
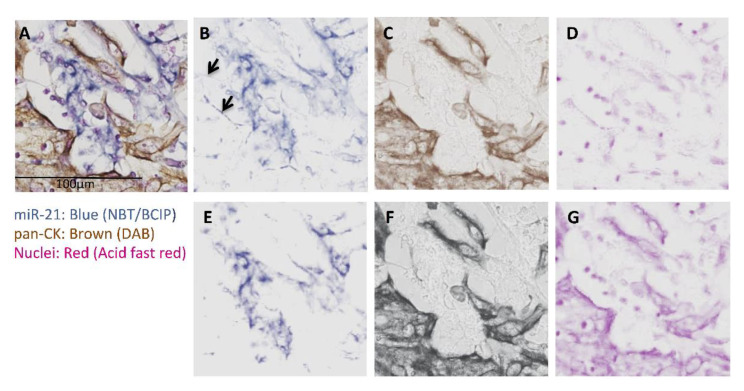
Color deconvolution of a representative image from a double stained section for miR-21 and pan-CK. (**A**) OSCC (oral squamous cell carcinoma) section stained for miR-21 (blue; NBT-BCIP), pan-CK (brown; DAB) and counter stained (red; acid fast red). Image A was color deconvoluted into individual color images: (**B**) (NBT-BCIP), (**C**) (DAB), and (**D**) (acid fast red). Color deconvolution using RGB vectors for black color instead for DAB (brown) for the same image took away non-NBT-BCIP signals ((**E**); indicated by arrows in (**B**), while overexposing brown (**F**) and red (**G**). Original magnification: 20×; scale bar: 100 µm. Vectors used: Nuclear fast red (NFR)-NBT-BCIP-DAB (NFR: R=0.350, G = 0.840, B = 0.408; NBT-BCIP: R = 0.677, G = 0.627, B = 0.384; DAB: R = 0.443, G = 0.598, B = 0.667), and NFR-NBT-BCIP-BLACK (NFR: R = 0.375, G = 0.827, B = 0.416; NBT-BCIP: R = 0.647, G = 0.649, B = 0.398; BLACK: R = 0.588, G = 0.578, B = 0.565).

**Figure 6:**
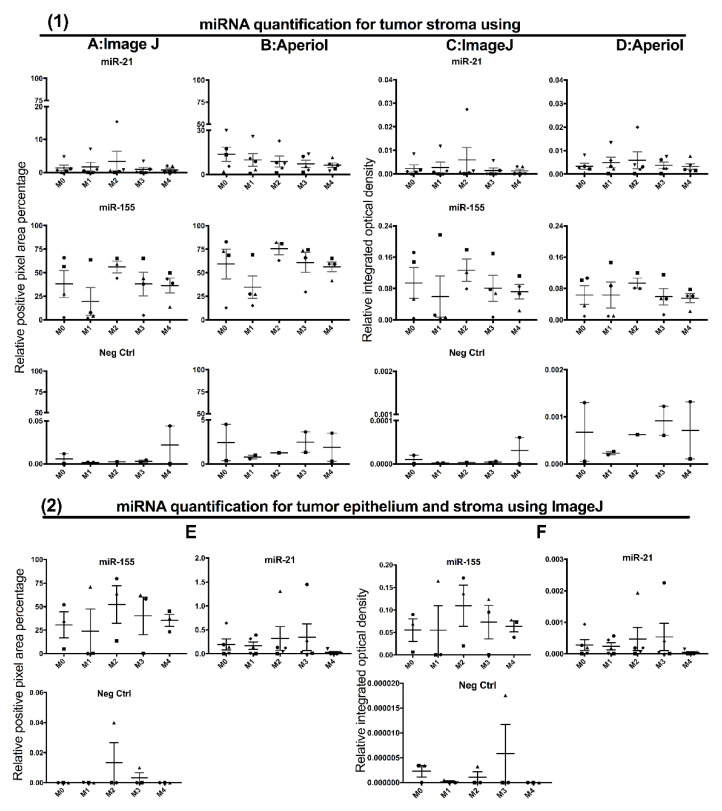
Graphs depicting quantification of miRs (miR-21 and miR-155) detected by different methods and using two different software. Means of positive pixel area percentage (PPAP, 1**A** and 1**B**) and integrated optical density (IOD, 1**C** and 1**D**) of miR signal in single ISH (control method: M0) and various combinations of double staining methods (M1-M4) were compared using paired Student´s t-test. No miR probe served as negative control tissue (Neg Ctrl). Two different types of image analysis software were used for the quantification: ImageJ (1**A**,1**C**,2**E**,2**F**) and Aperio (1**B** and 1**D**). In 2**E** and 2**F**, measurements include both tumor and stroma. Same symbols in the graphs indicate same tissue.

Quantitative assessment of miR signals using color deconvolution (Figure 5) in both ImageJ and AperioI software did not show significant differences in positive pixel area percentage (PPAP) or integrated optical density (IOD) between the control and the dual staining methods (Figure 6). Irrespective of the order of procedures in the combined double staining methods, and whether there was co-localization or not, both pan-CK and miRs were accessible to either binding antibody or probes, respectively, after their preceding stain (Figure 3).

### 3.3. Contribution of Noise to miR Signal in the Double Staining Methods

Overall, higher PPAP and IOD for the miR stains were detected by AperioI compared to ImageJ. The contribution of noise in the PPAP and IOD measurements of miR signals were assessed against the negative controls. PPAPs of 0.044–0.22% and 0.29–4.49%, and IODs of 0–0.2 × 10^−5^ and 1.3 × 10^−3^–5 × 10^−5^ were observed for ImageJ and AperioI, respectively (Figure 6(1)), while they were nearly absent when the whole image (tumor epithelium and stroma) was analyzed (Figure 6(2)). Of note, the background contributing to true signals in general comes from the dark fibers present in the images (arrow Figure 5B). Color deconvolution using RGB vectors obtained for black color instead for brown (DAB) took away this unspecific signal from darkly stained tissues (DAB saturated) and dark fibers (Figure 5E), and therefore the latter was used in the successive color deconvolution of the images.

Since all methods gave comparable results, the least technically challenging and most straightforward method (M3) was chosen for miR-204 staining of the OSCC cohort. AperioI was chosen for the quantification of the staining for the cohort due to the more convenient use of the deconvolution plugin and the automated quantification steps. 

### 3.4. Cohort Description and Prognostic Significance of Clinico-Pathological Parameters

**Figure 7:**
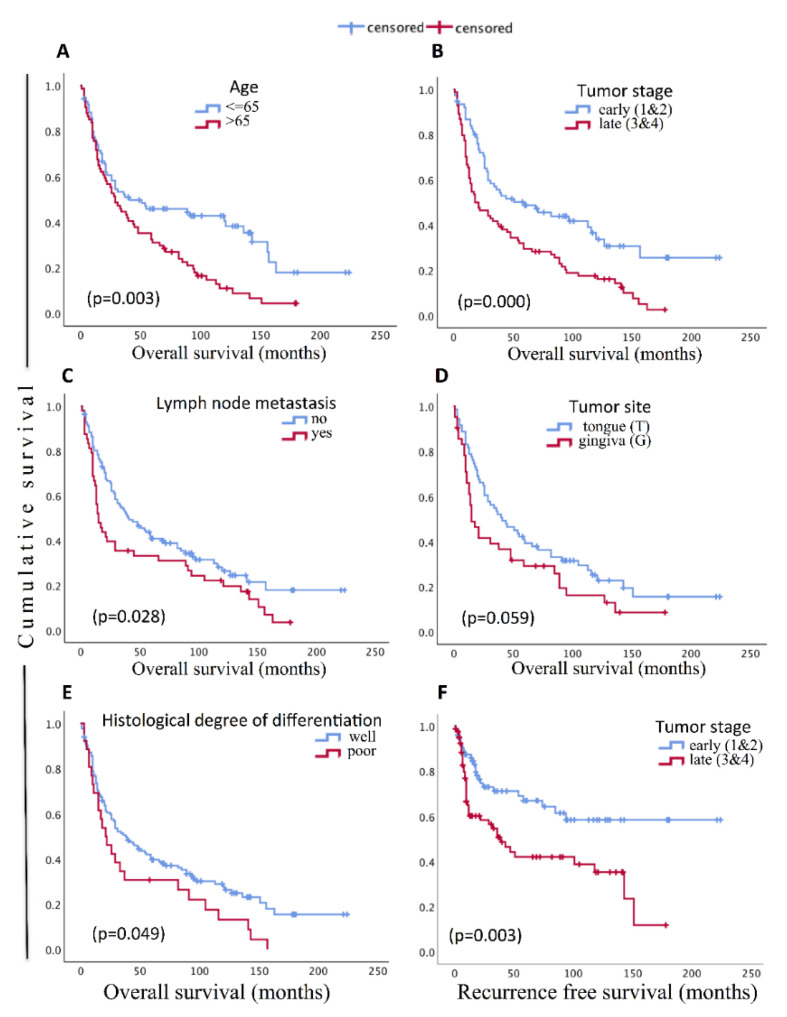
Kaplan–Meir plots of the survival functions (overall survival and recurrence-free survival) for sub-groups defined by different clinicopathological parameters and associated *p*-values (log-rank test). Only parameters with significant (*p* < 0.05) or near significant survival differences are shown.

Tests of associations in between clinicopathological parameters (Pearson’s Chi-square: Phi and Cramer’s V test ) showed significant association of WPI type 4 (*p* = 0.042), high tumor budding (*p* = 0.051), and late tumor stage group (3 and 4) (*p* = 0.027) with higher recurrence. Higher tumor budding (*p* = 0.019) and late tumor stage (*p* = 0.000) were associated with increased risk of lymph node metastasis. Localization of tumor in gingiva (*p* = 0.001) compared to tongue, and poor histological degree of differentiation (*p* = 0.007) were associated with late tumor stages. Tumor budding showed significant association with WPI (*p* = 0.001) and histological degree of differentiation (*p* = 0.004). Significant association between tumor stage and depth of invasion (*p* = 0.000), alcohol and gender (*p* = 0.005), smoking and gender (*p* = 0.001), smoking and alcohol (*p* = 0.000), age and gender (*p* = 0.001, higher proportion of males were in the age group >65), age and tumor site (*p* = 0.031), and age and smoking (*p* = 0.001, lower proportion of smokers in age group >65) were also observed. KM survival analysis of the clinicopathological parameters demonstrated significantly lower OS for lymph node metastasis and late tumor stage groups (Figure 7). For age groups, age group greater than 65 showed poorer OS compared to the younger group. OS was poorer with poorer histological degree of differentiation and when the site of tumor was the gingiva (Figure 7). Subsequent univariate Cox regression of the clinicopathological variables showed significant increase in relative risk of death for age group >65 years, late stage tumor, and lymph node metastasis. Tumor site in gingiva and poor histological degree of differentiation also showed significant increase in OS risk (Table 1). Multivariate Cox regression of the significant parameters in the univariate model revealed age and tumor stage as the independent predictors of the OS (Table 2). Only tumor stage showed significant association with RFS in the univariate analysis (Table 1). Late tumor stages approximately doubled the risk of recurrence compared to early tumor stages (Table 2).

### 3.5. Expression of miR-204 in NHOM and OSCC

Expression of miR-204 was very low in the connective tissue subjacent to normal oral mucosa, followed by significantly higher expression in the peritumoral regions, stroma of tumor front, and tumor center, in ascending order (Figure 4). However, both intertumor and intratumor heterogeneity in stromal expression of miR-204 was observed in OSCC samples (Figure 4). A subset of OSCCs expressed stromal miR-204 at a comparative level to the connective tissue of NHOM. Another subset of OSCC tissues showed higher expression of stromal miR-204, with higher expression in the stroma of the tumor center compared to the stroma of the tumor front. Nevertheless, concurrent expression of miR-204 in the stroma in tumor center and stroma in the tumor front was observed, with Spearman’s rho correlation revealing a very strong significant correlation (rs = 0.903; *p* = 0.000). A concomitant expression of miR-204 in the stroma and tumor cells was also observed (Figure 4A). Since qRT-PCR is widely accepted as the gold standard for miR expression analyses, to further validate that the double ISH-IHC method is sensitive and specific enough for quantification of miR-204 in FFPE samples we performed qRT-PCR on 10% of the cohort samples (16 randomly selected tissue samples). The correlation analysis revealed a significant correlation (Pearson r = 0.60; *p* = 0.01) between the two methods (Figure S1).

### 3.6. Prognostic Significance of Stromal miR-204 

A Pearson Chi-square test of miR-204 expression with the clinical variables showed significant association (*p* = 0.018) of miR-204 expression in the stroma of the tumor center with histological degree of differentiation. Near significant association (*p* = 0.052) with the same was observed for miR-204 expression in the stroma of the tumor front. For all other variables, no association was found. The test showed independence of age, gender, smoking, and alcohol with all the clinical variables, except for association of age with death (*p* < 0.001). Further association tests by Spearman rho correlation between miR-204 expressions with the clinical variables showed significant positive association of stromal miR-204 in the tumor center with histological degree of differentiation (r = −0.189; *p* < 0.05), i.e., increased histological differentiation was linked to higher miR-204 expression.

**Figure 8:**
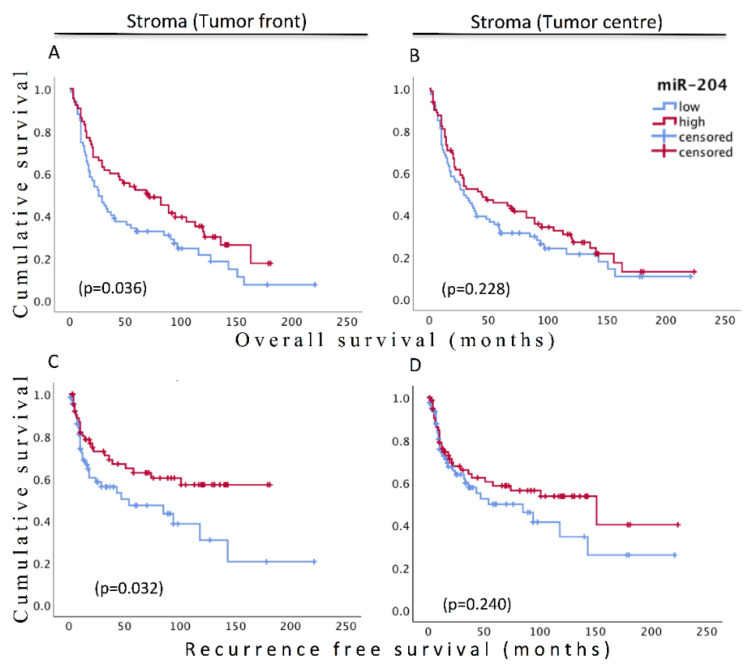
Kaplan–Meir survival plots (overall survival and recurrence-free survival) for low and high miR-204 expression groups in the stroma at the tumor front (**A**,**C**) and tumor center (**B**,**D**). *p* values are derived from log-rank test.

KM analysis of survival difference of the miR-204 high and miR-204 low group in the stroma in the tumor front predicted significantly better OS and RFS for high miR-204 group. Though statistical significance was not obtained, similar survival distribution appeared for the stromal miR-204 groups in the tumor center (Figure 8). In line with KM analysis, univariate Cox regression showed significant reduction in the relative risk of dying by 34.3% and recurrence by 46% for the miR-204 high group. miR-204 expression predicted similar outcomes in the multivariate model after adjusting for the age and tumor stage for OS and adjusting for stage in RFS (Table 2).

## 4. Discussion

Information on spatial location and distribution of miR in cancer tissues that is obtainable through miRs staining is more informative than the PCR-based methods and provides details on the role of cell- or tissue compartment-specific miRs in cancer. In addition, while staining of miRs informs us about their presence/absence in comparison to the normal adjacent tissues and hence their involvement in carcinogenesis, concomitant IHC for proteins can provide better mechanistic insights into cancer progression by miRs. Thus, dual staining of miR and protein provides superior information that might be used for a more accurate stratification of patients compared to individual detection of biomarkers. In our study, the double staining allowed us to accurately identify tumor stroma. Methods employing dual staining of miR and protein in same tissue section have been achieved recently in some studies, mostly using fluorochromes [13,35,36,37] or chromogens Nuovo [38]. A major drawback with the established methods is that the information on the influence of IHC on staining of miR by ISH is lacking, with most of the established methods being limited to IHC performed only after miR ISH. This study adds in the flexibility to stain low expressed miR or proteins after the ones with higher expression.

Different alternatives of combining miR ISH and pan-CK IHC were tested (Figure 1) and the effect of the steps involved in the double staining methods on staining outcome of individual stains were also examined. All the combined methods tested in this study could reliably detect pan-CK and miRs in a single FFPE tissue section. The primary concern was if DAB-based IHC would affect the miR ISH stain. Single miRs ISH with counter nuclear staining (method M0) was taken as a control method, and all other methods were compared to it. Method 1, miR ISH and subsequent IHC, was chosen to see if IHC would diminish or overlay ISH staining, and to find out whether primary antibodies in the IHC method can still find the antigen epitopes. Methods 2 and 3 were chosen to see if miR probe bound to miR or Fab bound to the DIG-linked probe in the miR-binding probe would be affected by steps involved in IHC. DAB reaction was introduced before NBT-BCIP reaction in method 2 to test if the DAB product would affect Fab-AP accessibility to NBT-BCIP reaction. DAB reaction was conducted at the final step in method 3, to see the similar effects of DAB reaction and/or product on miR stain as in method 1. Method 4 was chosen to see how IHC staining (DAB product) would affect miR accessibility, miR probe binding, and thus the final miR staining. On the other hand, the methods were also a guide as to how IHC staining is affected by steps involved in miR staining in the combined double staining methods. 

All the protocols used showed absence of nonspecific miR signals by using different negative controls. One such negative control for the miR-specific probes is to use scramble oligonucleotide probes with no target-binding site. In this study a scramble oligonucleotide was tested at five different temperatures (48 °C, 50 °C, 51 °C, 53 °C, and 55 °C). The scramble oligonucleotide showed positive staining at 48 °C. Therefore, despite lacking any target-binding site, a scramble oligonucleotide may not be a universal control probe. A more suitable scramble control is the one with a melting temperature the same as that of miR specific probe. In ISH, melting temperature of a target oligonucleotide probe largely determines its specificity. The higher the hybridization temperature, the higher the specificity, but this may compromise the signal. On the contrary, a lower hybridization temperature may increase signal, but can result in cross-hybridization to a similar sequence, causing increases in unspecific binding or the noise. Having no miR target probe in a negative control in miR ISH is a test for antibody specificity, and a measure of efficient blocking. Failure in specific antibody binding, and insufficient blocking, both result in a false positive signal. Specific tissues can also be used as controls based upon established staining results, but the results can be the function of sensitivity of the methods used. Here we used miR-21 on OSCC samples; miR-21 is a well-established tumor stroma-specific miR in OSCC and it is not expressed in normal oral mucosa; however, it is also not expressed in a subgroup of oral cancers [13]. Therefore, inter-individual and intra-tissue heterogeneity that may result in heterogeneous staining outcomes should also be considered when evaluating the controls. 

Noise and signal are completely unavoidable in any staining methods. Color deconvolution to blue from darkly stained DAB was observed previously in a pioneering color deconvolution study by Ruifrok and Johnston [39]. We have been able to remove a major part of the noise by demonstrating that vectors obtained for black color instead for brown (DAB) can take away signal from saturated stain and dark fibers. Another way to avoid noise would be to exclude such tissue compartments from annotation. In our observation, noise can also occur from inefficient blocking, low hybridization temperature, high antibody and substrate concentration, incubation and/or reaction temperature, and time. We also found a nonspecific binding of anti-DIG-Fab to stroma in normal oral mucosa. Perhaps this is inherent to some tissues or an outcome of harsh pre-treatments.

ImageJ is an open source program for image analysis. Free availability of ImageJ and its plugins is a major advantage over AperioI in image quantification. However, there are a few limitations. The first is related to annotation. Distance and area measurement require additional steps such as setting up the scale measurements and command for the measurements. In addition, annotations need to be permanently saved within the pictures if they are to be analyzed or revisited later. The annotation shortcomings of ImageJ can be compensated by annotation using freely available software such as NDP.view2 (Hamamatsu) or AperioI. Secondly, unless one can program ImageJ to record and install macros for the steps involved in quantification such as annotation selection, deconvolution, threshold setting, and quantification, for automated analysis and batch feeding, all the steps need to be carried out one at a time, and pictures fed individually, which takes a significant amount of time. All these steps are automated in AperioI and take less time, but the plugin required for the quantification requires paid licensing. In addition, introducing a new vector (color) in ImageJ color deconvolution plugin is technically challenging, while it is user friendly in AperioI once one has the plugin. 

We further showed that the combined method gives comparable quantifiable results to the gold standard method of qRT-PCR, in addition to having the advantage of showing cellular localization of the miR of interest. Although the aim of this study was not to investigate the expression of miR-21 and miR-155, and their staining was done on only a limited number of cases here, our results further confirm their pattern of expression in OSCC. MiR-21 was previously shown to be expressed exclusively in the tumor stroma of OSCC and to correlate with poor prognosis [13]. It was also shown to have a biological importance in development of tongue squamous cell carcinoma by inhibiting cancer cell apoptosis [40] and regulation of the expression of multiple target genes important for cancer progression such as phosphatase and tensin homolog (PTEN) and programmed cell death protein 4 (PDCD4) that regulate the radiosensitivity and sensitivity to cisplatin in OSCC [41]. MiR-155-5p has been previously shown to be expressed in both tumor cells and the associated stroma of OSCC, as confirmed by our staining pattern as well. However, the focus has been on its biological role in tumor cells and its expression was linked to EMT-associated OSCC progression [42].

A major limitation of our combined ISH and IHC double staining methods is that our methods are limited to antibodies that can be used with a proteinase K treatment for antigen retrieval in FFPE tissues. We have not tested antibodies that require other retrieval procedures. However, frozen tissues could be used for miR ISH and protein IHC without any pre-treatment [37]. In this study, the focus was on the quantification of the miR, while the visualization of protein (pan-CK) by IHC was used to differentiate between tumor and stromal compartments. In the context of a high degree of heterogeneity of both tumor cells and tumor stroma, the combinatorial detection of miR and one or more marker proteins at the same time will provide additional advantages for further cell identification and characterization. Although challenging, this methodological approach can also be further developed to determine co-expression of a miR and a potential target protein and to quantify the results of both ISH and IHC (including in addition to one miR, a second and/or a third protein) in the case of co-localization. The proteinase K digestion step used to improve tissue access in the protocols presented here may damage or remove some protein antigens, therefore the prehybridization digestion step must be particularly optimized and evaluated for a further, broader applicability.

After establishing a combined ISH-IHC method and digital quantification for the miRs, we tested the method in a clinically relevant biomarker quest on an OSCC cohort. Age, tumor stage, lymph node metastasis, and recurrence are the well-recognized prognostic indicators in OSCC. In this cohort we found tumor stage and age to be independent prognostic indicators of survival. Similarly, tumor stage was an independent prognostic indicator of recurrence. Lymph node metastasis and poor histological degree of tumor differentiation correlated with reduced survival in the univariate analysis. As reported in previous OSCC studies, we found that higher tumor stage correlated with reduced recurrence-free survival [13,34]. In this cohort the 5-year survival was 40%, which is comparable to some other previous reports [43,44]. Tumor site in the gingiva showed poorer survival compared to tumors in the tongue, probably due to its proximity to the bone leading to early bone invasion and the association with late tumor stages.

This study indicates a dynamic and complex regulation of miR-204 in the stroma of OSCC. Previous studies in different cancers including OSCC showed overall reduced expression of miR-204 [15,16,17]. Higher levels of miR-204 have been associated with better survival in several cancer types [16,17,20,22,23], including OSCC [25]. These findings are in line with our study, which shows association of higher expression of miR-204 with better survival, albeit the association is only for stromal expression, not whole tumor. Contrary to the previous study on whole tumor tissues, the present study finds an increased expression of miR-204 in the stroma in a subset of OSCC tissues when compared to NHOM. Nevertheless, previous studies on breast cancer [17] and OSCC [25] also exhibited a subset of tumors in which miR-204 expression was higher than a subset of the normal counterpart tissues. Meanwhile, cell context-based tumor suppressive and oncogenic dual function of miR-204 in pancreatic cancer cells lines [45], and miR-204 expression-dependent metastasis of cancer cells in vivo in mice, have been shown earlier [17].

Moreover, compared to NHOM, we also found a relatively higher expression of miR-204 in the matched peritumoral connective tissue of the high miR-204-expressing OSCC, often subjacent to epithelial dysplastic changes. This may indicate that alterations of miR-204 occur early in carcinogenesis and evolve concomitant to cancer progression, at least in a subset of OSCC. These findings might point towards increased miR-204 expression as a protective mechanism that the stroma develops as a reaction to the progressive changes in the epithelium. This is the first study to show that, similar to the antitumor effect when expressed in tumor cells, high expression of miR-204 in stromal cells is also detrimental to tumor progression. Hence, this study on patient material, together with previous studies, suggests a prognostic benefit of higher expression of miR-204 in cancers including OSCC.

## 5. Conclusions

The approach of using pan-CK to exclude epithelium, especially difficult when identifying single cells or poorly differentiated cancer cells, is important in studying tumor stroma in tumors of epithelial origin. The double staining and the quantification methods demonstrated in this study can be used in integrative biomarker studies based on the same tissue sections that can provide superior information to single biomarkers. We have applied the method in studying stromal miR-204 in OSCC and found miR-204 as a prognostic indictor of survival and recurrence-free survival in OSCC.

## Figures and Tables

**Table 1:** Univariate estimates of the risks of the clinicopathological parameters by Cox regression.

Parameters	N (%)	Overall Survival		Recurrence Free Survival	
		*p*-Value	HR (95% CI)	*p*-Value	HR (95% CI)
miR-204_TF		0.04		0.036	
Low	67 (41.9)		1		1
High	65 (40.8)		0.657 (0.44–0.98)		0.56 (0.33–0.96)
miR-204_TC		0.234		0.245	
Low	79 (49.4)		1		1
High	79 (49.4)		0.804 (0.56–1.15)		0.75 (80.46–1.22)
Age (years)		0.003		0.333	
≤65	85 (53.1)		1		1
>65	74 (46.3)		1.73 (1.20–2.48)		0.27 (0.78–2.08)
Gender		0.296		0.191	
Female	58 (36.3)		1		1
Male	102 (60.5)		1.22 (0.84–0.18)		1.42 (0.84–2.38)
Alcohol		0.137		0.51	
Low-Normal	51 (31.9)		1		1
Moderate-High	35 (21.9)		1.47 (0.89–2.43)		1.23 (0.63–2.55)
Smoking		0.44		0.287	
No	49 (30.6)		1		1
Yes	75 (46.9)		1.18 (0.78–1.79)		1.37 (0.77–2.46)
Tumor site		0.063		0.591	
Tongue	71 (44.4)		1		1
Gingiva	42 (26.3)		1.50 (0.98–2.30)		1.10 (0.77–1.56)
Stage		0.001		0.004	
Early (1&2)	76 (47.5)		1		1
Late (3&4)	84 (52.5)		1.90 (1.32–2.75)		2.11 (1.27–3.50)
T stage		0.005		0.032	
T1			1		1
T2		0.002	1.90 (1.13–3.21)	0.107	1.82 (0.88–3.77)
T3		0.104	1.63 (0.90–2.929	0.178	1.76 (0.77–4.02)
T4		0	2.47 (1.50–4.07)	0.003	2.86 (1.42–5.77)
Lymph node		0.031		0.108	
No metastasis	112 870)		1		1
Metastasis	48 (30)		1.51 (1.04–2.2)		1.52 (0.91–2.54)
Distant metastasis		0.113		0.662	
No	139 (86.9)		1		1
Yes	21 (13.1)		0.677 (0.42–0.10)		1.19 (0.54–2.61)
Depth of invasion		0.21		0.261	
Superficial (<4mm)	41 (25.6)		1		1
Deep (≥4mm)	46 (28.7)		1.376 (0.84–0.27)		1.51 (0.74–3.10)
Tumor budding score		0.647		0.19	
Low (<5 buds)	72 (45)		1		1
High (≥5 buds)	58 (36)		1.097 (0.74–1.63)		1.43 (0.84–2.43)
Histological degree of differentiation					
Well diff	72 (45)	0.053	1	0.392	1
Poor diff	58 (36)		1.55 (0.99–2.4)		1.32 (0.70–2.47)
Worst pattern of invasion					
Type 1–3	19 (11.9)	0.578	1	0.187	1
Type 4	111 (69.4)		1.47(0.89–2.43)		1.99 (0.72–5.50)

**Table 2:** Multivariate Cox regression for the significant parameters from the univariate model and miR expressions.

Parameters	N (%)	^a^ Overall Survival		^b^ Recurrence Free Survival	
		*p*-Value	HR (95% CI)	*p*-Value	HR (95% CI)
miR-204_TF		0.048		0.033	
Low	67 (41.9)		1		1
High	65 (40.8)		0.668 (0.45–1.00)		0.55(0.32–0.95)
miR-204_TC		0.26		0.193	
Low	79 (49.4)		1		1
High	79 (49.4)		0.812 (0.56–1.17)		0.72 (0.44–1.22)
Age (years)		0.004			
≤65	85 (53.1)		1		
>65	74 (46.3)		1.80 (1.20–2.70)		
Stage		0.005		0.004	
Early (1&2)	76 (47.5)		1		1
Late (3&4)	84 (52.5)		1.78 (1.12–2.67)		2.11 (1.27–3.50)
Lymph node		0.108			
No metastasis	112 870)		1		
Metastasis	48 (30)		1.057 (1.04–2.2)		

^a^ Adjusted for age and tumor stage; ^b^ Adjusted for tumor stage.

## Data Availability

Data supporting reported results will be made publicly available if the manuscript is accepted for publication.

## Supplementary Materials

The following are available online at https://www.mdpi.com/2072-6694/13/6/1307/s1, Figure S1: QQ correlation plot between the levels of miR-204 detected by qRT-PCR (y-axis) and by dual ISH-IHC (method 3—x-axis) in 16 FFPE tissue sections, Table S1: Clinical and pathological characteristics of the OSCC cohort.
