# Supplementary material for: Combined In Situ Hybridization and Immunohistochemistry on Archival Tissues Reveals Stromal microRNA-204 as Prognostic Biomarker for Oral Squamous Cell Carcinoma
Source: Cancers (Basel). 2021 Mar 15;13(6):1307. doi: 10.3390/cancers13061307 (PMC8002032; doi:10.3390/cancers13061307)
Supplement: Supplementary file 1 [file cancers-13-01307-s001.pdf]

cancers

MDPI

Article

Combined in situ Hybridization and Immunohistochemistry on Archival Tissues Reveals Stromal microRNA-204 as Prognostic Biomarker for Oral Squamous Cell Carcinoma

Saroj Rajthala, Harsh Dongre, Himalaya Parajuli, Anjie Min, Elisabeth Sivy Nginamau, Arild Kvalheim, Stein Lybak, Dipak Sapkota, Anne Christine Johannessen and Daniela Elena Costea

Supplemantary Materials:

Table S1. Clinical and pathological characteristics of the OSCC cohort.

Parameters	N (%)	Parameters	N (%)
Gender		Histological degree of differentation	
Female	58 (36.3) (median range 74)	Well diff	72 (45)
Male	102 (60.5) (median range 62)	Poor diff	58 (36)
Alcohol		Recurrence	
Low-Normal	51 (31.9)	No	95 (59.4)
Moderate-High	35 (21.9)	Yes	65 (40.6)
Unkown	36 (22.5)		
Smoking		Depth of invasion	
No	49 (30.6)	Superficial(<4 mm)	41 (25.6)
Yes	75 (46.9)	Deep(≥4 mm)	46 (28.7)
Unkown	36 (22.5)	Unquantifiable	73 (45.6)
Tumor Site		Tumor budding score	
Tongue	71 (44.4)	Low (<5 buds)	72 (45)
Gingvia	43 (26.9)	High (≥5 buds)	58 (36)
Buccal	20 (12.5)	Unquantifiable	30 (18.8)
Floor of mouth	18 (11.3)		
Overlapping	8 (5)		
		Worst pattern of invasion	
T stage		Type 1	2 (1.3)
T1	43 (26.9)	Type 2	3 (1.9)
T2	47 (29.3)	Type 3	14 (8.8)
T3	26 (16.3)	Type 4	111 (69.4)
T4	44 (27.5)	Unquantifiable	30 (18.8)
N stage		Distant metastatic progression	
N0	112 (70)	No	
N1	22 (13.7)	Yes	139 (86.9)
N2	26 (16.3)	Lung	21 (13.1)
N3	0	Skin	9 (5.6)
M stage		Other	5 (3.1)
M0	156 (97.5)	(bone, brain, adrenals)	7 (4.4)
M1	4 (2.5)		
Stage			
Stage Ⅰ	43 (26.9)		
Stage Ⅱ	37 (23.1)		
Stage Ⅲ	25 (15.6)		

Stage Ⅳ	57 (35.6)	[illegible]

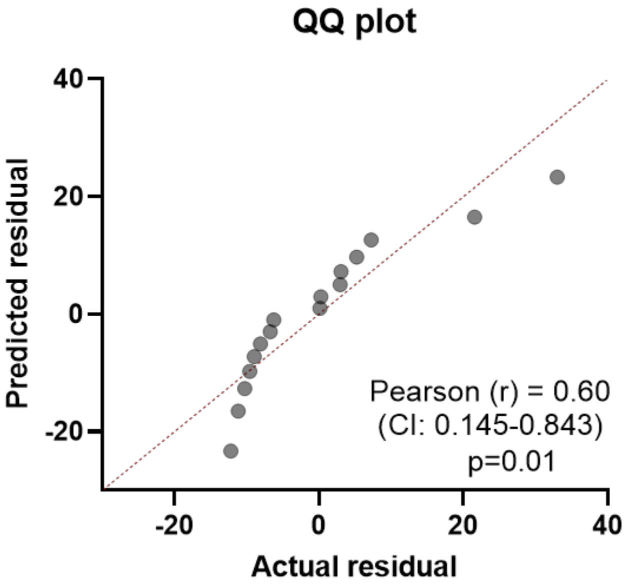

Figure S1. QQ correlation plot between the levels of miR-204 detected by qRT-PCR (y-axis) and by dual ISH-IHC (method 3—x-axis) in 16 FFPE tissue sections.
